# Case Report: Solitary fibrous tumor of the pelvis associated with Doege–Potter syndrome

**DOI:** 10.3389/fonc.2026.1864354

**Published:** 2026-10-06

**Authors:** Xiaolong Tan, Weikai Dong, Wenli Chang, Xi Chen

**Affiliations:** 1 Department of Gastrointestinal Surgery, Binzhou Medical University Hospital, Binzhou, Shandong, China; 2 Cardiovascular Medical Center, Nanjing Drum Tower Hospital, The Affiliated Hospital of Nanjing University Medical School, Nanjing, Jiangsu, China; 3 Department of Pathology, Binzhou Medical University Hospital, Binzhou, Shandong, China; 4 Department of Emergency Surgery, Binzhou Medical University Hospital, Binzhou, Shandong, China

**Keywords:** Doege-Potter syndrome, insulin-like growth factor 2, non-islet cell tumor hypoglycemia, pelvic tumor, solitary fibrous tumor

## Abstract

**Introduction:**

Solitary fibrous tumor (SFT) is an uncommon mesenchymal spindle cell neoplasm (incidence ~1.4 per million). Although most SFT occur in the thoracic cavity, they can also arise in the retroperitoneum, pelvis, head, and neck. While the majority exhibit benign behavior, approximately 5% are associated with paraneoplastic syndromes, most notably Doege–Potter syndrome (DPS). DPS is characterized by non-islet cell tumor hypoglycemia (NICTH) resulting from tumor hypersecretion of high-molecular-weight insulin-like growth factor 2 (IGF2), which saturates insulin and IGF receptors causing refractory, life-threatening hypoglycemia. Due to nonspecific presentation and low incidence, DPS secondary to pelvic SFT is frequently misdiagnosed, underscoring the need for heightened clinical awareness.

**Case report:**

A 56-year-old man presented to the neurology department with acute loss of consciousness, aphasia, and urinary incontinence. He had a 3-year history of hypertension but no diabetes mellitus. Initial laboratory tests showed a blood glucose of 4.76 mmol/L; however, during hospitalization, he developed recurrent severe hypoglycemia, with a nadir glucose of 1.6 mmol/L. Further biochemical evaluation during fasting hypoglycemia revealed undetectable insulin (<0.2 mIU/L) and low C-peptide (0.14 ng/mL). Contrast-enhanced abdominopelvic CT demonstrated a 17.8 cm heterogeneously enhancing pelvic mass causing bladder compression and left ureteral displacement. The patient declined preoperative biopsy. Preoperative euglycemia was maintained with continuous intravenous dextrose infusion, and open en bloc surgical resection was performed. Histopathology and immunohistochemistry (positive for STAT6, CD34, and CD99; Ki-67 8%) confirmed an atypical solitary fibrous tumor. Postoperatively, hypoglycemic symptoms resolved completely, and the patient was discharged on postoperative day 6 without further hypoglycemic episodes.

**Conclusion:**

Pelvic SFT with DPS frequently presents with recurrent hypoglycemia or altered consciousness rather than local compressive symptoms. Early recognition of non-islet cell tumor hypoglycemia (NICTH) and prompt cross-sectional imaging are essential for accurate diagnosis. Complete surgical resection is definitive and results in full resolution of hypoglycemia. Given the unpredictable biological behavior of SFT, long-term follow-up is essential. Heightened clinical awareness may improve diagnostic accuracy and patient outcomes.

## Introduction

Solitary fibrous tumor (SFT), first described by Klemperer and Coleman in 1931 (1), is an uncommon soft tissue neoplasm arising from mesenchymal spindle cells. SFTs occur in adults of all ages, with no apparent sex predilection (2). Although most SFT arises in the thoracic cavity, it has also been reported in the retroperitoneum, head and neck, and pelvis (3). The 2020 WHO classification of soft tissue tumors categorizes SFT as an intermediate tumor with malignant potential (4). Most SFT does not recur locally or distantly. The 5- and 10-year disease-specific survival rates are approximately 75% and 55%, respectively (5). The tumor may eventually become clinically apparent through compressive symptoms caused by its growth. Several histological criteria have been recognized as effective for assessing malignancy in solitary fibrous tumors. These factors encompass augmented tumor size, mitotic index, cellular density, occurrence of bleeding or necrosis, and nuclear pleomorphism (2, 6). Approximately 5% of SFT exhibit ectopic hormone secretion sufficient to induce hypoglycemia. Doege-Potter syndrome (DPS) is a rare and complex paraneoplastic syndrome characterized by non-islet cell tumor hypoglycemia (NICTH) (7).

NICTH results from activation of insulin receptors by high-molecular-weight insulin-like growth factor 2 (IGF-2), which is produced by tumors of epithelial or mesenchymal origin (8). In this condition, high-molecular-weight IGF-2 overstimulates insulin and IGF-I receptors, leading to refractory hypoglycemia (9). Although glucocorticoids, glucose supplementation, and dietary modification provide temporary relief of hypoglycemia (10), s complete surgical resection of the underlying tumor remains the definitive treatment for SFT-associated NICTH.

Herein, we report a 56-year-old man who presented with recurrent severe hypoglycemia with neuroglycopenic manifestations caused by a large pelvic solitary fibrous tumor. Surgical resection effectively resolved these symptoms. We also discuss the clinical presentation, management, and pathological characteristics of this condition. This report aims to raise awareness of this syndrome.

## Case report

A 56-year-old man presented to the neurology department on September 14, 2024, with acute loss of consciousness, aphasia, and urinary incontinence. His medical history included a 3-year history of hypertension managed with extended-release oral nifedipine; there was no history of diabetes mellitus, hepatitis, or autoimmune disease. Admission laboratory tests revealed a blood glucose of 4.76 mmol/L (reference range, 3.9–6.1), glycated hemoglobin (HbA1c) of 6.0% (reference range, 4–6%), potassium of 3.11 mmol/L (hypokalemia; reference range, 3.5–5.3), and calcium of 2.12 mmol/L (hypocalcemia; reference range, 2.2–2.7). Cranial CT and electroencephalography (EEG) showed no acute abnormalities. On physical examination, the patient was overweight (BMI, 29.01 kg/m²) and had severe hypertension (179/110 mmHg). Neurological assessment revealed normal muscle tone and absent pathological reflexes; complete motor evaluation was limited by impaired consciousness.

During hospitalization, recurrent hypoglycemia led to transfer to the endocrinology department for specialized management. Biochemical evaluation during fasting hypoglycemia revealed a glucose level of 1.6 mmol/L (reference range, 3.9–6.1), insulin <0.2 mIU/L (undetectable; reference range, 2.6–24.9), and C-peptide 0.14 ng/mL (reference range, 1.1–4.4). Glutamic acid decarboxylase antibodies, insulin autoantibodies, cortisol, adrenocorticotropic hormone (ACTH), thyroid-stimulating hormone (TSH), and free T_3_ were within normal limits. Tumor markers (AFP, CA19-9, CEA) were unremarkable (**Table 1**). After abdominal ultrasound showed bilateral hydronephrosis, contrast-enhanced abdominopelvic CT revealed a 17.8 cm heterogeneously enhancing pelvic mass causing bladder compression and left ureteral displacement (**Figure 1**). The patient was transferred to gastrointestinal surgery for definitive resection. Abdominal examination post-transfer identified a palpable nontender immobile mass with smooth contours in the lower abdomen/pelvis. The patient declined percutaneous biopsy and MRI. Integrative analysis of clinical, biochemical, and radiological findings suggested solitary fibrous tumor (SFT), liposarcoma, or paraganglioma as leading differential diagnoses.

**Table 1:** Laboratory blood values during spontaneous hypoglycemia.

Test	Results	Reference range
Serum blood glucose	1.6	3.9–6.1(mmol/L)
Serum insulin	<0.2	2.6–24.9(mIU/L)
C‐peptide	0.14	1.1–4.4(ng/ml)
ACTH	6.07	1.1-13.2(pmmol/L)
TSH	3.04	0.27-4.2(mIU/L)
Free T3	4.21	2.8-7.1 (pmmol/L)
Free T4	14.85	12-22 (pmmol/L)
Cortisol	281.1	171-536(nmol/L)
Angiotensin II	26.54	25-250(pg/ml)
Aldosterone	22.60	70-300(pg/ml)
Renin	13.20	3.11-41.2 (μIU)

ACTH, adrenocorticotropic hormone; TSH, thyroid-stimulating hormone.

**Figure 1:**
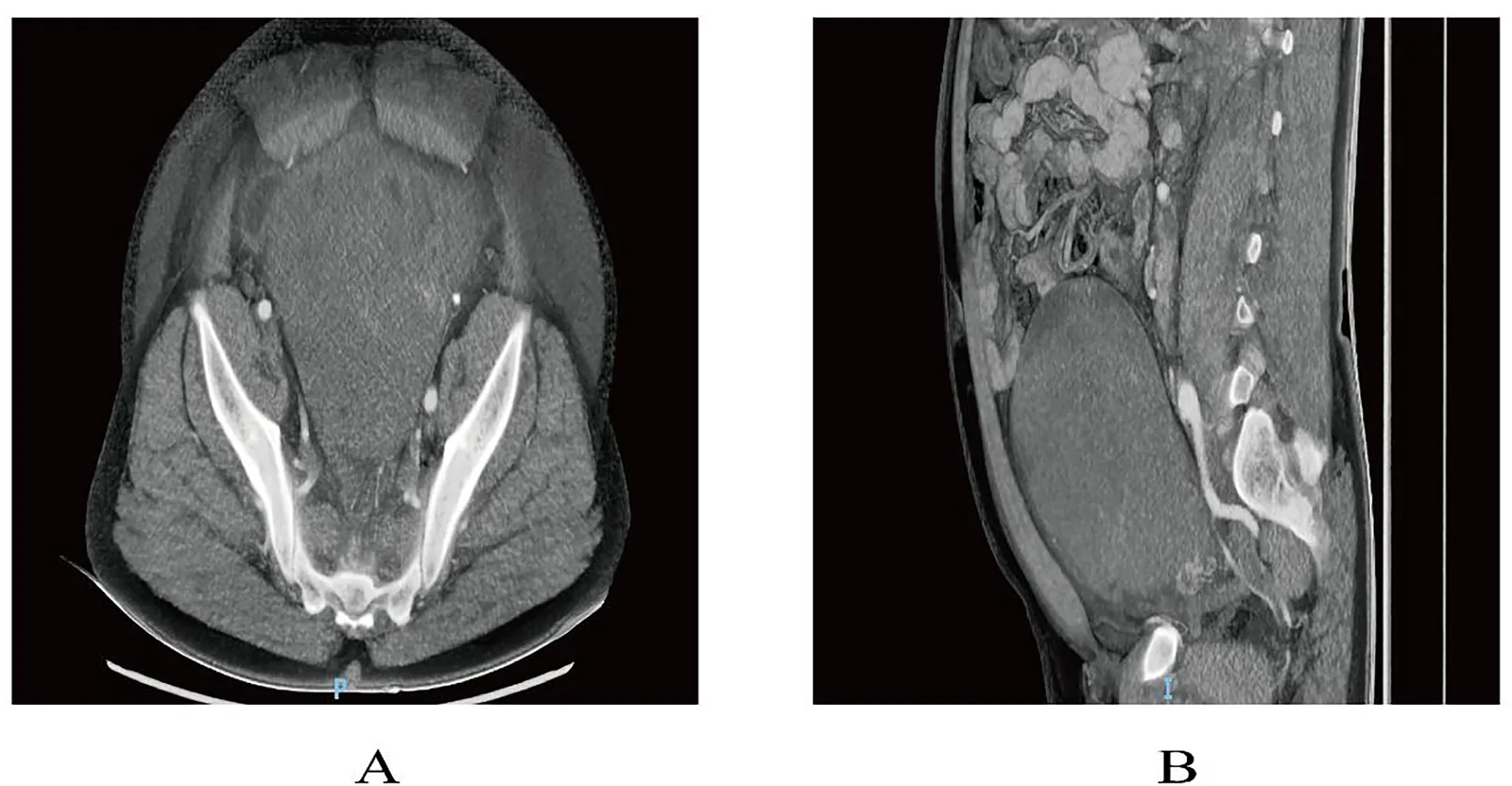
CT scan of a large abnormal smoothly contoured pelvic mass, cross-sectional and coronal plane images. [**(A)** Axial plane, **(B)**: sagittal plane. No signs of perifocal infiltration in adjacent structures are present].

Dietary management alone was ineffective in controlling hypoglycemia. Capillary blood glucose was monitored every 6 hours, with supplemental intravenous glucose infusion, to maintain preoperative euglycemia. Multidisciplinary team (MDT) review identified the following key surgical risks: massive hemorrhage from the hypervascular tumor, refractory hypoglycemia triggered by intraoperative tumor manipulation, ureteral injury due to close tumor-ureter adherence, and hemodynamic instability after abrupt tumor resection. Preoperative optimization included volume resuscitation and glucose infusion before open en bloc resection. Intraoperatively, the tumor occupied the retrovesical space, displacing the bladder cephalad toward the umbilicus and precluding successful ureteral stenting. The well-encapsulated mass was completely excised along the peritumoral fascial plane without injury to adjacent organs. The procedure lasted 2 hours, with an intraoperative blood loss of 400 mL. **Figure 2** shows the resected specimen with prominent feeding vessels and an intact, glistening capsule.

**Figure 2:**
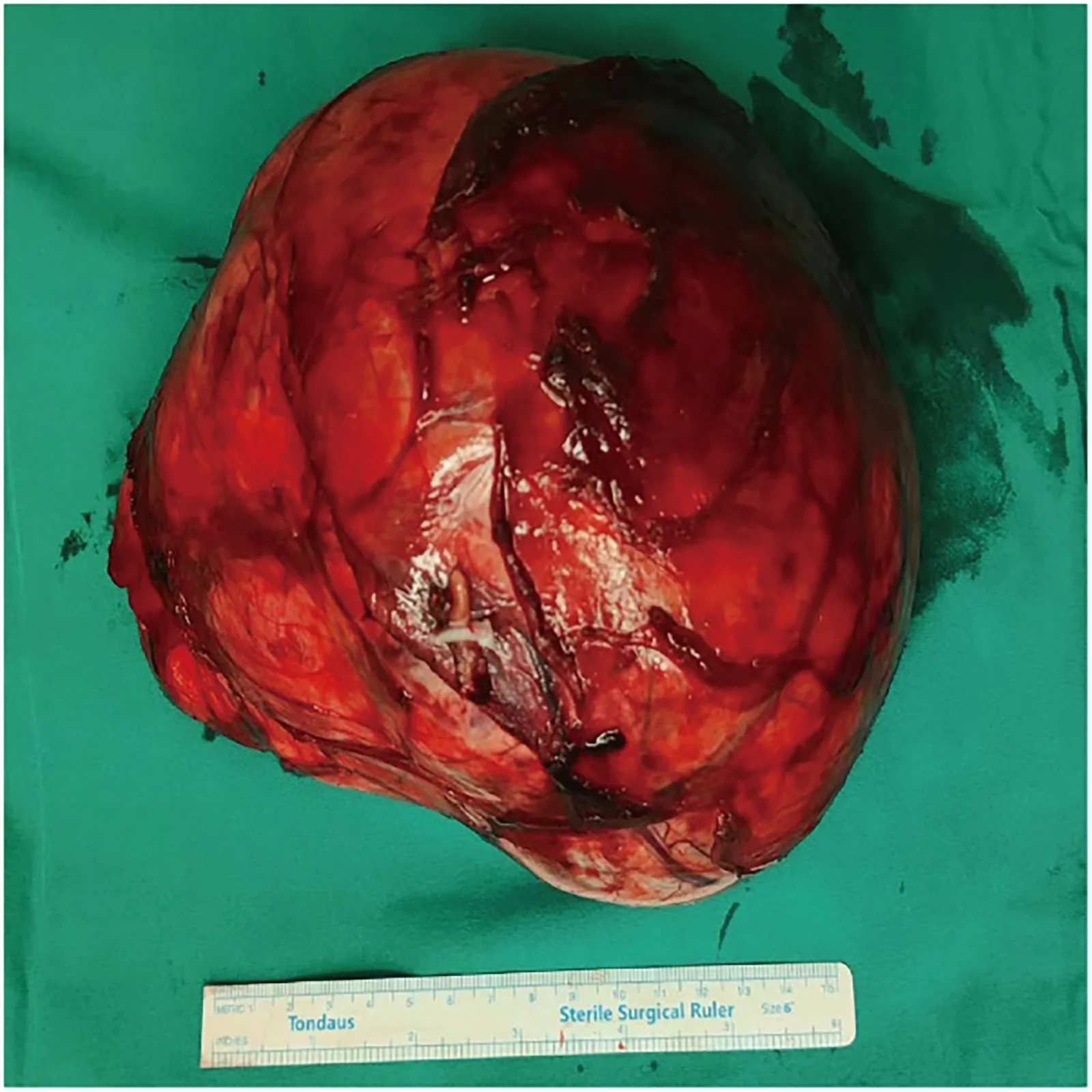
Macroscopic appearance of the tumor.

Pathological examination revealed a SFT measuring 16.8 × 13 × 13 cm, with a grayish-white, multinodular cut surface and a pseudo-capsule. Histologically, the tumor displayed increased cellularity, marked cytologic atypia, and readily identifiable mitotic figures (>4 per 10 high-power fields in hypercellular areas). Necrosis was not identified. According to current risk stratification models, these findings were consistent with an atypical SFT and classified as high risk. Immunohistochemically, the tumor cells exhibited diffuse nuclear STAT6 positivity, a diagnostic hallmark of SFT, along with diffuse expression of vimentin, CD34, and CD99. β-catenin showed cytoplasmic staining without nuclear accumulation. Desmin was focally and weakly positive (1+), and p16 was partially expressed. The Ki-67 proliferation index was approximately 8%. The tumor was negative for CD117, DOG-1, S-100, SMA, MDM2, and CDK4 (**Figure 3**). Postoperative normalization of blood glucose levels was achieved, with no recurrence of hypoglycemic episodes. The patient was discharged on postoperative day 6 in stable clinical condition. At the 6-month telephone follow-up, the patient reported no recurrent hypoglycemia. However, he declined further in-person clinical and imaging follow-up. Given the high-risk nature of the tumor, the lack of imaging surveillance represents a significant limitation, and the true recurrence status remains unknown.

**Figure 3:**
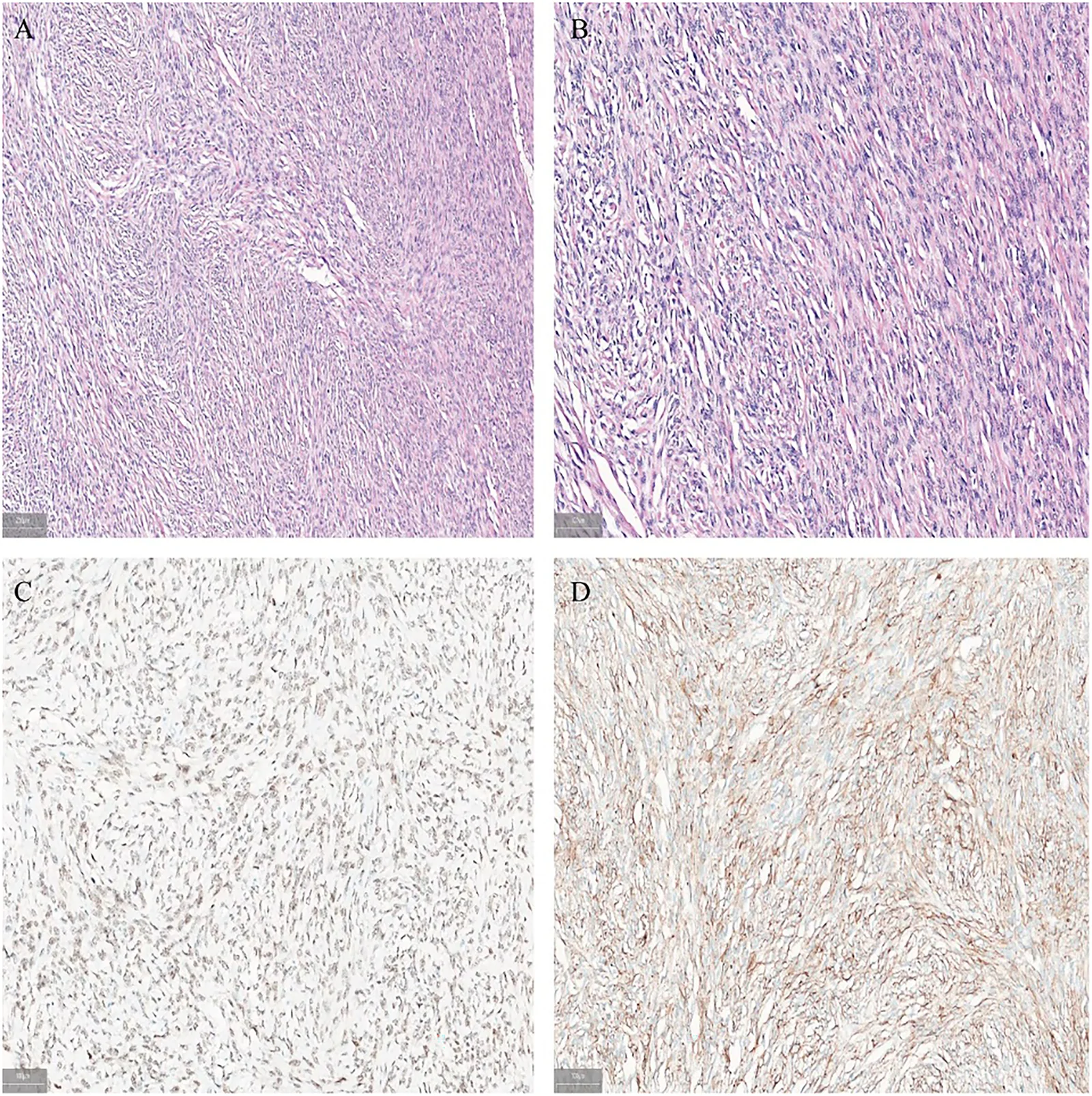
The representative hematoxylin-eosin **(A, B)** photomicrographs of a solitary fibrous tumor. **(A)** magnification ×100, **(B)** magnification ×200. **(C)** STAT6 expression, immunohistochemical staining method, magnification ×200. **(D)** CD34 expression, immunohistochemical staining method, magnification ×200.

## Discussion

Solitary fibrous tumor (SFT) is uncommon mesenchymal neoplasms with an estimated incidence of 1.4 per million (11). Although SFT are classically described in the thoracic cavity (12), data from the French Sarcoma Group suggest that abdominal and pelvic sites may be more frequent (13). Paraneoplastic hypoglycemia occurs in fewer than 5% of SFT and constitutes DPS (14). Since 1991, only 17 pelvic SFT associated with DPS have been reported (3, 9, 15–29) (**Supplementary Table S1**). Including the present case, the 18 patients had a median age of 62 years (range, 28–83), a balanced sex distribution, and a mean tumor diameter of 17 cm. The most common presentations were hypoglycemia-related symptoms and abdominal complaints. Notably, pelvic cavity often permits occult tumor growth, delaying diagnosis until the tumor reaches a large size, as observed in our patient.

When evaluating adults presenting with recurrent severe hypoglycemia, differentiating hyper insulinemic from hypo insulinemic etiology represents the essential first step. In our patient, severe fasting hypoglycemia (1.6 mmol/L) with undetectable insulin and low C-peptide, ruling out insulinoma or exogenous insulin administration, and suggested NICTH. NICTH is most commonly caused by tumor secretion of high-molecular-weight insulin-like growth factor 2 (big IGF-2) (30), which circulates in an abnormal form that binds IGF-binding proteins less avidly (31), thereby increasing free IGF availability and activating insulin receptors to suppress hepatic glucose output (32). Hypersecretion and elevated bioavailability of the big IGF2 result in the clinical manifestation of NICTH.

Notably, not all patients with SFT and DPS exhibit high serum IGF-2 level. In the literature we examined, the serum IGF-2 level was normal in certain instances (22, 24). By binding to tissue insulin receptors, big IGF-2 accelerates peripheral glucose utilization and suppresses the secretion of growth hormone and insulin. At the same time, IGF-1 secretion is directly regulated by growth hormone (33). Consequently, the IGF-2/IGF-1 ratio, rather than the absolute IGF-2 level, is used clinically as a diagnostic marker for NICTH; an IGF-2/IGF-1 ratio greater than 10:1 is considered diagnostic (34). The diagnosis of Doege-Potter syndrome was considered highly likely based on recurrent hypo insulinemic hypoglycemia, the presence of a large solitary fibrous tumor, and complete resolution of hypoglycemia after tumor resection. However, IGF-1, IGF-2, and big IGF-2 levels were not available, which represents a limitation of this report.

To date, only 17 cases of pelvic SFT with DPS have been reported in the international literature (**Supplementary Table S1**). The wide and deep pelvic cavity allows SFT to grow silently, often reaching a large size before causing local compressive symptoms (mean reported diameter, 17 cm; 17.8 cm in the present case). Consequently, patients typically present with systemic neuroglycopenic symptoms, such as sudden loss of consciousness, aphasia, or convulsions, rather than a palpable pelvic mass, which may lead to delayed diagnosis or misdiagnosis as a neurological disorder. Clinicians should therefore consider an occult extra pancreatic tumor in patients presenting with unexplained impaired consciousness and hypoglycemia.

Surgical resection is the optimal and paramount treatment for SFT (13). NICTH can cause life-threatening neuroglycopenic injury. Maintaining stable preoperative blood glucose levels requires continuous intravenous infusion of high-concentration glucose (often requiring 10%–20% concentration) and hourly capillary blood glucose monitoring (35). In cases of severe refractory hypoglycemia, short-term glucocorticoids or recombinant growth hormone may serve as bridging therapy by suppressing tumor release of bioactive big IGF-2 and stimulating gluconeogenesis (36, 37). Pelvic SFT are typically hyper vascular tumors. Resection of a large pelvic mass carries a high risk of catastrophic intraoperative bleeding. Evidence suggests that super selective transcatheter arterial embolization (TAE) performed 24–48 hours before surgery is a valuable adjunctive measure (16). Preoperative TAE can significantly reduce tumor blood supply, decrease intraoperative blood loss, and improve surgical field visualization. Owing to the deep pelvic containment, giant SFT inevitably compress adjacent pelvic structures, leading to obstructive hydronephrosis via ureteral displacement, severe vesical compression, or rectal obstruction. Surgical planning must incorporate multidisciplinary strategies, such as preoperative ureteral stent placement (29), to safeguard pelvic retroperitoneal structures. In this case, precise circum-tumoral dissection along fascial planes enabled successful en bloc resection without collateral organ damage. Complete tumor removal led to instantaneous postoperative normalization of blood glucose and permanent resolution of DPS symptoms, confirming the curative role of surgery.

Histopathology and immunohistochemistry (IHC) are indispensable for definitive SFT diagnosis. Solitary fibrous tumors consist of spindle-shaped or oval cells interspersed with staghorn-shaped blood veins within the tissue (38). The diagnosis relies on immunohistochemistry, most importantly diffuse nuclear STAT6 positivity, which reflects the NAB2-STAT6 fusion gene and has become the diagnostic hallmark (39). Although CD34 is positive in up to 80% of SFT (40), its expression may be lost in high-grade or recurrent tumors (41). BCL2 was identified as being favorably expressed in the majority of SFT (42).

The differential diagnosis for a large pelvic mass is broad, and distinguishing a SFT from other entities requires a comprehensive multidisciplinary approach incorporating radiological, biochemical, and histopathological findings. Well-differentiated and dedifferentiated liposarcomas, common in the retroperitoneum and pelvis, can be excluded by the absence of MDM2 and CDK4 expression (4). Paragangliomas, which often mimic the hyper vascular appearance of SFT on cross-sectional imaging, are ruled out by negative immunohistochemical staining for neuroendocrine markers such as chromogranin and synaptophysin, along with normal biochemical catecholamine profiles. Extra-gastrointestinal stromal tumor (GIST) is excluded by the lack of CD117 (c-kit) and DOG1 expression (43), whereas neurogenic tumors (e.g., schwannomas) are excluded by negative S100 and SOX10 staining (44). Similarly, leiomyosarcoma and other smooth muscle tumors are ruled out by the absence of SMA, desmin and h-caldesmon. Ultimately, the exclusion of these lineage-specific markers, together with strong and diffuse nuclear STAT6 expression, a highly sensitive and specific surrogate for the pathognomonic NAB2-STAT6 gene fusion, definitively confirms the diagnosis of SFT.

Although most SFT exhibit indolent clinical behavior, long-term biological outcomes remain unpredictable. Pasquali, S et al (45) and Demicco et al (46) stratified recurrence risk based on mitotic rate, cellularity, and cellular proliferation. To substantiate the pathological diagnosis of atypical SFT, we retrospectively applied the Demicco risk stratification model to the present case. The score was calculated as follows: age ≥55 years (1 point), tumor size ≥15 cm (3 points), mitotic count >4 per 10 HPF (2 points), and absence of tumor necrosis (0 points). The total score of 6 falls into the high-risk category, which is in perfect accordance with the histopathological assessment. This formal classification is concordant with the pathological diagnosis of atypical SFT and underscores the need for long-term surveillance. Unfortunately, our patient declined scheduled imaging follow-up, although a telephone follow-up at six months confirmed no further hypoglycemic episodes. This represents a limitation of the present report.

This case highlights the clinical imperative of screening for pelvic extra-pancreatic neoplasms in adults presenting with unexplained hypo insulinemic hypoglycemia. It demonstrates that even giant, hyper vascular pelvic SFT causing life-threatening paraneoplastic syndromes can achieve complete biochemical and oncologic cure through proactive perioperative glycemic stabilization and meticulous en bloc resection.

## Conclusion

In summary, this case contributes to the limited literature on pelvic SFT with Doege-Potter syndrome by illustrating that severe hypoglycemia may be the first manifestation of an occult pelvic mass, that a clinical diagnosis of NICTH can be established even when specialized IGF assays are unavailable, and that complete surgical resection remains the cornerstone of management. The high-risk features further emphasize the importance of long-term follow-up.

## Data Availability

The original contributions presented in the study are included in the article/**Supplementary Material**. Further inquiries can be directed to the corresponding author.
