# Supplementary material for: Case Report: Solitary fibrous tumor of the pelvis associated with Doege–Potter syndrome
Source: Front Oncol. 2026 Oct 6;16:1864354. doi: 10.3389/fonc.2026.1864354 (PMC13680167; doi:10.3389/fonc.2026.1864354)
Supplement: Supplementary file 1 [file Table1.docx]

Supplement Table 1 Cases of Pelvic Solitary fibrous tumors (SFTs) with Doege-Potter syndrome (DPS) reported from 1991 to 2025

| Author | Year | Age | Gender | System | Treatment | Tumor size（cm） | Mitotic rate | Ki-67(%) | CD34 | CD99 | Vimentin | IGF-II | Bcl-2 | STAT6 | S-100 | SMA | Outcome | Follow-up time(month) |
| --- | --- | --- | --- | --- | --- | --- | --- | --- | --- | --- | --- | --- | --- | --- | --- | --- | --- | --- |
| Chen, Sijing et al. | 2018 | 37 | Female | a pelvic mass, severe hypoglycemia | Resection | 16 | 14/10HPF | NA | + | + | + | NA | - | NA | - | - | Recurrence | 43 |
| Tanaka, Motoki et al. | 2022 | 71 | Female | genital bleeding | Resection | 20 | 1/10HPF | 20% | + | NA | NA | + | NA | + | NA | NA | Disease-free | 24 |
| Yuza, Kizuki et al. | 2020 | 46 | Female | persistent hypoglycemia, a giant pelvic mass | Resection | 17 | 2/10HPF | NA | + | + | + | + | + | + | NA | NA | Disease-free | 24 |
| Mao, Wangjia et al. | 2024 | 82 | Female | loss of consciousness | Laparoscopic resection | 12 | 4/10HPF | 15% | + | NA | NA | NA | + | + | - | - | NA | NA |
| Wada, Yukiko et al. | 2019 | 72 | Male | hypoglycemic attack | Resection | 15 | 7/10HPF | NA | + | + | NA | + | + | + | - | - | Disease-free | 12 |
| Sheikh, Khalid et al. | 2024 | 66 | Male | confusion, lethargy | Oral  glucocorticoid+ Resection | 23.5 | NA | NA | + | NA | NA | NA | NA | + | NA | NA | Disease-free | 12 |
| Dahiya, Divya et al. | 2013 | 48 | Male | gradually increasing lower abdominal lump, abdominal discomfort | Resection | 20 | NA | NA | + | NA | NA | NA | NA | NA | NA | NA | Disease-free | 6 |
| Ishihara, Hiroki et al. | 2016 | 72 | Male | loss of consciousness | Resection | 9 | NA | <5% | + | NA | + | + | + | NA | + | NA | Disease-free | 9 |
| Thiruchelvam, Nirosshan et al. | 2015 | 70 | Female | loss of consciousness | Oral  glucocorticoid | 20 | NA | NA | NA | NA | NA | NA | + | NA | NA | NA | Disease-stable | 12 |
| Hata, Tatsuo et al. | 2012 | 83 | Male | loss of consciousness | Resection | 12 | NA | NA | + | NA | NA | NA | + | NA | NA | NA | Recurrence | 12 |
| Pinho Dos Santos, Diana et al. | 2021 | 72 | Female | loss of consciousness | Resection | 10 | 0/10HPF | <10% | + | - | NA | NA | + | NA | - | NA | Disease-free | 3 |
| Wagner, Sigrid et al. | 2009 | 28 | Male | left flank pain | Resection | 18 | NA | 3% | + | + | NA | NA | + | NA | - | - | NA | NA |
| Nagase, Takeshi et al. | 2005 | 60 | Male | hypoglycemic crisis | Resection | 22 | 10-20/10HPF | NA | + | NA | + | + | + | NA | - | - | NA | NA |
